# Malaria Vaccine Development: The Need for Novel Approaches: A Review Article

**Published:** 2018

**Authors:** Shima MAHMOUDI, Hossein KESHAVARZ

**Affiliations:** 1. Pediatric Infectious Disease Research Center, Tehran University of Medical Sciences, Tehran, Iran; 2. Center for Research of Endemic Parasites of Iran (CREPI), Tehran University of Medical Sciences, Tehran, Iran; 3. Dept. of Medical Parasitology and Mycology, School of Public Health, Tehran University of Medical Sciences, Tehran, Iran

**Keywords:** Malaria, Vaccine candidates, Different approaches

## Abstract

**Background:**

Although rigorous efforts have substantially decreased the malaria burden through decades, it still threatens the lives of millions of children. Development of an effective vaccine can provide important approach in malaria control strategies. Unfortunately, development of an effective vaccine for *falciparum* malaria has been hindered by the extreme complexity of malaria parasite biology, complex and diverse parasite genomes, and immune evasion by the parasites as well as the intricate nature of the parasites infection cycle. The aim of this review was to discuss the different approaches to malaria vaccine development until now.

**Methods:**

Scientific databases, including MEDLINE (via PubMed) and SCOPUS were searched up to 30 Jan 2017 and the articles regarding malaria vaccine development were taken into examination.

**Results:**

Several strategies for malaria vaccine development including pre-erythrocytic vaccines, antibody-based subunit vaccines, vectored vaccines, whole sporozoite vaccines, genetically Attenuated parasites and sporozoite subunit vaccine, erythrocytic vaccines, sexual stage vaccine, transmission-blocking vaccine as well as synthetic peptides and conjugate vaccine has been introduced. However, the success has been limited thus far.

**Conclusion:**

Although development of malaria vaccine over the past 70 year has been continued, the discovery, development, and licensing of a malaria vaccine formulation, which meets safety, affordability, accessibility, applicability, and efficacy has not yet been achieved.

## Introduction

Malaria remains as one of the leading causes of morbidity and mortality, especially in the tropics. Although rigorous efforts have substantially decreased the malaria burden through decades, it still threatens the lives of millions of children (1).

In 2015, 212 million people developed clinical malaria infection, mostly in resource-poor tropical areas of the world and in particular sub-Saharan Africa and 428000 people died of the disease (2). Of those infected, around 500000 succumb to malaria, with children under the age of 5 and pregnant women being the most susceptible.

The necessity to develop a vaccine against malaria has been emphasized from the identification of the parasite in 1897. In 1897, Ronald Ross discovered the mosquito (vectors) that transmit the disease. Further, only the female *Anopheles* mosquito can transmit the parasite. However, aiming to develop a highly effective malaria vaccine has led to the use wide range of new approaches (3). The emergence of resistant parasites and vectors has caused to concentrate on other controls achievements including vaccine (4).

Development of an effective vaccine can provide important approach in malaria control strategies (5). Unfortunately, development of an effective vaccine for *falciparum* malaria has been hindered by the extreme complexity of malaria parasite biology, complex and diverse parasite genomes, and immune evasion by the parasites as well as the intricate nature of the parasites infection cycle.

Generally, the majority of the available vaccines are divided into following categories: attenuated versions of pathogen microbes, killed microbes or protein subunits or conjugate vaccines (3).

Although vaccination has several successful strategies in reducing of some diseases incidence, development of vaccines for malaria has remained a challenge because of antigenic variability and the requirement of T-cell immunity for protection (5). Parasite vaccines usually face the challenges. They generate low immunity and they mostly need to proper adjuvants and most of the selected malaria antigens as vaccine candidates show significant genetic polymorphism they are the targets of natural immunity (3).

There has been considerable progress in development of malaria vaccines. Several factors should be considered for development of each new vaccine: pathogen life cycle and epidemiology, immune control and evasion, antigen candidate and vaccine formulation and preclinical/clinical results (6). One of the major obstacles to vaccine development is complex life-cycle of the parasite and the variability of antigens within each stage (5).

There are several malaria vaccine candidates which were undergone different phase of clinical trials; however, until now there was not a good candidate with appropriate efficacy. Because the parasite has three different life stages, there are three distinct vaccines approaches based on sporozoites, sexual and asexual forms.

In this review, we discuss the different approach of the malaria vaccine development until now. This review of the malaria literature highlights current approach of the current malaria vaccine development and discusses their status and challenges.

## Methods

Scientific databases, including MEDLINE (via PubMed) and SCOPUS were searched up to 30 January 2017. There was no beginning data limitation and the articles regarding malaria vaccine development were taken into examination.

### Results and Discussion *Plasmodium* parasites life-cycle

The three stages in the *Plasmodium* life cycle can be divided into two distinct categories: in the pre-erythrocytic and erythrocytic asexual and gametocytes reproduction occurs in the intermediate host’s body, and in the sexual stage *sexual* reproduction occurs in the mosquito vector gut (7).

Sporozoites of *Plasmodium* are inoculated to the subcutaneous of humans by the bite of infected Anopheles mosquitoes. After minutes, they enter to liver via bloodstream. Then, after 6–15 d tissue merozoites enter bloodstream and begin asexual blood schizogony as well as gametocyte production. After that, the gametocytes enter the midgut of vector mosquitoes and finally, sporozoites are formed due to the sexual stage of parasite development. In each stage of *Plasmodium* life cycle, various antigens can be introduced into the human body and they can stimulate host immune system (8).

**Fig.1:**
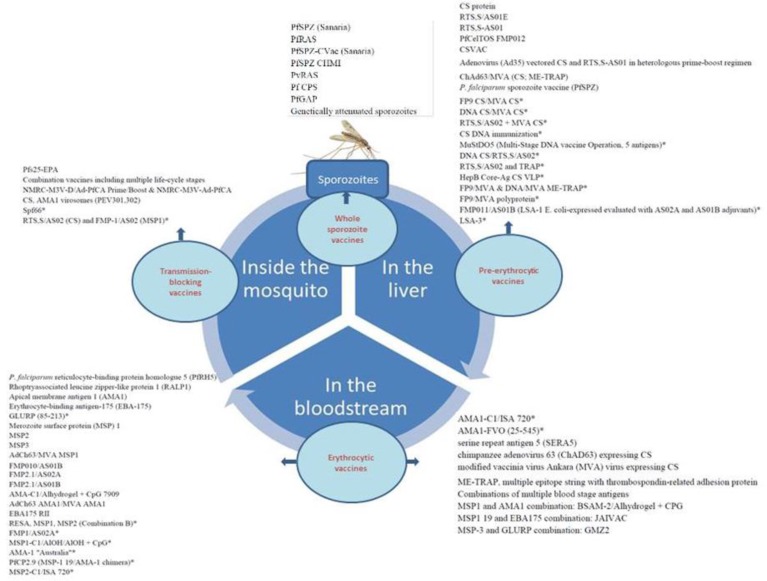
Life cycle of the malaria parasite and breaking of the cycle by major vaccine groups *Discontinued/inactive

The pre-erythrocytic, erythrocytic stages and transmission-blocking vaccines (TBVs) against asexual stages are considered as the main targets of *P. falciparum* parasites for vaccine development. However, recently, the old approach of whole parasite vaccination has improved rapidly (Fig.1).

### Immunity against the Malaria Parasite

Numerous innate and adaptive immune mechanisms are involved for control and elimination of malaria due to infecting of different cell types and wide range of its localizations (9). Understanding the immune responses to the malaria parasites is considered as the crucial goal for development of vaccines.

Protective immunity against malaria requires interaction between both innate and adaptive immunity (dendritic cells, NK cells, B cells, CD4^+^ and CD8^+^ T cells). On the other hand, to avoid being eliminated by the host immunity, the parasite introduce many escape strategies (9, 10) (Table 1).

**Table 1:** Parasite immune escape strategies

***Avoid recognition by complement and prevent complement activation system***
Prevent parasite elimination, inhibiting phagocyte function and reducing phagocyte numbers
Decrease DC functions
Antigenic diversion
Epitope masking
Avoid recognition by antibody
Prevent the action of neutralizing antibodies
Poor or limited B-cell memory
Poor or no antibody response by inducing immunological tolerance
Avoid T-cell recognition
Poor T-cell response
Anergy and/or T-cell exhaustion
Negative regulation of immune responses
Production of suppressor factors
Smoke-Screen strategy by cross-reactivity
Homologies with Host Proteins

### Pre-erythrocytic (PE) vaccines Radiation-attenuated sporozoites

Development of modern malaria vaccine stems from rodents, primates, and human volunteers immunization with irradiated sporozoites since 1960s (11, 12).

In 1967, immunization of mice was showed with radiation-attenuated *Plasmodium berghei* sporozoites (11). In 2002, the gamma radiation-attenuated sporozoites inside infected Anopheles mosquitoes lead to a complete protection in human. This irradiation approach had two major flaws: it was not cost-effective and not practical on a large scale (13).

### Monoclonal antibodies and recombinant DNA methodologies

In the early 1980s, technologies such as monoclonal antibodies and recombinant DNA methodologies were introduced. Although several antigens had been cloned and sequenced (14, 15), their role in protective immunity was only assessed by testing homologues in experimental infections.

### Antibody-based subunit vaccines

A drawback of this method is the lack of inflammatory cytokines induced by most subunit vaccine the need for adjuvant for immunogenicity and these vaccines could not target the liver-stage malaria parasite. In the future, several factors including evaluation of additional targets may increase the efficacy of antibody-based subunit vaccines such as PfCSP. In addition, finding of both PfCSP and non-PfCSP alike antigens for capturing of natural parasite heterogeneity and developing vaccines which induce long-lasting protection should be considered (16).

PE vaccine could prevent invasion of hepatocytes by sporozoite or kill parasites within infected hepatocytes. Efforts to identify novel sporozoite antibody targets have been difficult. Novel antigen selection will need to draw and combinations of multiple antigens may act synergistically to improve protection.

“RTS, S/AS01”, a subunit vaccine, elicit CD4 T cell and antibody responses to the *P. falciparum* circumsporozoite protein, or PfCSP (17). Although this vaccine has been recommended for licensure by EMEA and is the first vaccine to undergo large-scale phase 3 evaluations in seven African countries, it's efficacy in infants is relatively low, and the vaccine apparently will not meet the goal of malaria eradication by itself (18).

### Vectored vaccines

Vectored vaccines were introduced in attempts to induce higher efficacy. Chimpanzee adenoviruses (ChAds) encoding the thrombospondin-related adhesion protein (TRAP) pre-erythrocytic antigen was used to prime an immune response and then it was boosted by another viral vector, modified vaccinia virus Ankara (MVA) encoding the same TRAP insert (19, 20). This particular prime-boost approach first discovered in malaria introduces much higher T-cell responses than single vector immunization.

### Whole sporozoite vaccines

Most recently, the old approach of whole parasite vaccination has progressed rapidly through three major technologies including Radiation-attenuated *P. falciparum* sporozoites (RAS), Chloroquine Prophylaxis and Sporozoites (CPS) and Genetically Attenuated Parasites (GAP) (21).

Three different strategies have been employed to induce protective responses against liver stages. Irradiated sporozoites have the ability to invade liver cells and initiating liver stage development including expression of new proteins. They do not undergo nuclear division and formation of liver stage merozoites (22). It is more possible that PfSPZ-based vaccines will be introduced in WHO malaria vaccine roadmap during the next two to three years (23).

### Genetically Attenuated Parasites (GAP)

GAPs are generated by gene deletions, genome edition or stage-specific toxins expression (21).

Deletion of two pre-erythrocytic genes (p52-/p36-) in the NF54 strain resulted in introducing of the first generation *P. falciparum* GAP (24). The first experiments in mice demonstrated that single gene deletions using p52-/p36 genetically attenuated parasites (GAP) could elicit long-lasting protection mediated by CD8+ T cells (23, 24). GAP parasites induced larger and broader CD8+ T cell responses and long-lasting effector memory CD8+ T cells than RAS parasites in mice (25, 26).

Recently, triple KO Pf GAP (p52-/p36-/SAP-) (27) and alternative *P. falciparum* GAP vaccine is based on b9/slarp deficient sporozoites (28) was introduced. RAS Sanaria® PfSPZ-GA1 is another genetically attenuated strain, with deletions of the b9 and slarp genes (28). GAP parasites recognize antigens in both the late liver stages and blood and elicit stage transcending immunity. This may lead to identification of novel approach for development of a multi-stage subunit vaccine (29).

### Chloroquine Prophylaxis and Sporozoites (CPS)

Another approach for human immunization with whole sporozoites combines the bites of non-attenuated fully infectious *P. falciparum*-infected mosquitoes with chloroquine prophylaxis (CPS) (30). Chloroquine prevent blood stage development, while it has no effect on sporozoites and liver stages (31). The CPS procedure is safe and well tolerated and requires for fewer bites of fully infective mosquitoes to elicit 100% efficacy compared to RAS immunization.

The probable reasons for its higher efficacy could be due to expression of more liver stage antigens and development of larger antigen loads than RAS. In addition, the rout of immunization by bite might induce more potent immune responses than IV immunization with *P. falciparum* RAS (32)

The PfSPZ Vaccine approach has been adapted for CPS. In addition, nonirradiated sporozoites can be administered with chloroquine prophylaxis (called Sanaria® PfSPZ-CVac) (23).

### Sporozoite subunit vaccination

The circumsporozoite protein (CSP), a sporozoite surface antigen, was introduced in the era of subunit vaccine development for malaria (12).

Although subunit vaccines can eliminate the risk of reversion and have good safety compared to inactivated or attenuated pathogen vaccines, they have weakness in immune stimulation. Nanoparticles are introduced in the era of subunit vaccine development and they can enhance the efficacy of these vaccines by extending of the antigen release and circulation time (33).

However, production of a full-length CSP was unsuccessful, several substitute constructs such as R16tet32, R32tet32, R48tet32, and R32LR were produced (34). The RTS, S vaccine is another subunit vaccine based on the CSP repeat and C-terminal regions formulated with hepatitis C surface antigen in AS adjuvants (23).

### Erythrocytic vaccines

Erythrocytic or blood-stage vaccines (Fig.1), aim to stop the rapid invasion and asexual reproduction of the parasite in the red blood cells. Effective vaccination with blood stage would prevent complications such as renal failure and all manifestation of severe malaria in pregnancy. *P. falciparum* erythrocyte membrane protein 1 (PfEMP1), expressed on the surface of infected erythrocytes, was first cloned in 1995 and was considered as the prime target for an anti-complication vaccine.

At present, several clinical trials on blood-stage antigens including apical membrane antigen 1 (AMA1), erythrocyte-binding antigen-175 (EBA-175), glutamate-rich protein (GLURP), merozoite surface protein (MSP) 1, MSP2 and MSP3 and serine repeat antigen 5 (SERA5) have performed (35). Until now, two phases II clinical trial have been conducted with PfAMA-1. One of these trials, AMA1 vaccine with AS02 adjuvant was applied in 383 Malian children. Another vaccine trial with AMA-13D7 and AMA1-FVO and ALhydrogel in 279 children was studied; however, no significant impact on clinical malaria episodes was found (36). Although MSP1 vaccine could produce protective response s in Monkeys, the Trial phase II this vaccine with AS02 adjuvant in Kenyan children indicated no significant impact on clinical malaria (37).

The possible reason might be due to the extremely polymorphic nature of the vaccine structures (38–48). The combination malaria vaccine consists MSP-1, MSP-2 and RESA antigens with Montanide adjuvant completed phase 2 trial on 120 children 5–9 yr old. The vaccine showed no efficacy against clinical malaria (4).

None of the assays/models has been proven as a surrogate of protection and no blood-stage vaccines have shown strong efficacy in a large phase II (or III) trial. Therefore, discovery of novel antigen is highly recommended.

### Sexual stage vaccine

Only a few sexual stage vaccines in preclinical studies were introduced and only phase 1 trial is underway. This vaccine is intended to block the life-cycle of *Anopheles* mosquitoes.

Previous clinical trials of sexual stage vaccines involved ookinete antigens Pfs25 from *P. falciparum* and Pvs25 from *P. vivax*. However, these studies were not successful due to unacceptable reactogenicity most likely related to the adjuvant (49).

### Transmission-blocking vaccine (TBV)

TBVs is an indirect approach to a vaccine and target sexual stage antigens of Plasmodium (50). One TBV candidate vaccine is the *Pfs*25-EPA. Evidence shows that Pfs48/45 is a good TBV candidate (50); however, it's development have several challenges such as high epitopes variability for protective antibodies and the appropriate folded recombinant protein and improper secondary structure due to incorrect cysteine connectivity (50).

### Combination vaccines

Since developing of highly effective vaccines based on any single life cycle stage was not successful, introducing of effective vaccines which include antigens from more than one stage of the parasite's life cycle is highly suggested (3).

Several antigens including Py and Pb antigens alone or in combination with CSP have been selected for combination vaccine studies. It has been reported that PyLISP1 and PySLARP, PbLISP1, PbSLARP and PbPF3D7conferred significantly greater protection than that seen with CSP (51).

### Synthetic peptides and conjugate vaccine

Synthetic peptides have been exploited also as an approach for the development of a malaria vaccine. SPf66, a peptide-based candidate vaccine containing antigens from the blood stages of malaria linked together with an antigen from the sporozoite stage, and is targeted mainly against the blood (asexual) stages showed apparent efficacy in new world monkeys and humans (52); however, independent field efficacy trials in Africa and Asia failed to demonstrate good protection.

Another synthetic peptide candidates vaccine derived from two *P. falciparum* blood-stage antigens, MSP-1 and MSP-2, was introduced in Phase I studies in Sri Lanka, but it was in doubt that it was able to induce significant levels of antibodies against the parent blood-stage epitopes and fixed merozoites or not (53).

Synthetic vaccine based on peptide-tetanus toxoid conjugated was introduced (53). Due to the low potency of first-generation DNA vaccines (54), heterologous prime-boost immunization approaches with non-replicating viral vectors have been exploited (55).

### Other approaches

Parasite toxins (GPI-based) and adhesion ligands such as PfEMP1 are considered as another approach to vaccine development. Use of parasite antigens, such as the Var2 protein preferentially expressed in the placenta is another vaccine development method to prevent malaria in pregnancy.

The development of passive immunization by gene therapy could be considered as another potential promise particularly for pathogens which can evade from current vaccination strategies due to antigenic variability (56).

Vectored immunoprophylaxis has been demonstrated as another approach which was effective in a host of animal models for the prevention of malaria (56).

## Conclusion

Although development of malaria vaccine over the past 70 year has been continued, the discovery, development, and licensing of a malaria vaccine formulation, which meets safety, affordability, accessibility, applicability, and efficacy has not yet been achieved.

The march toward development of new approaches will require continued efforts and resources. Some approaches based on reverse vaccinology in order to search the genome for finding the best protective antigens is highly recommended to developing a multicomponent vaccine with full and long-lasting protection.

Since a malaria vaccine would be an important tool for prevention and treatment, further study on using of alternative parasite targets and vaccination strategies, and evaluating of other vaccine candidates in the pipeline through vaccine trials, are highly recommended.
